# Genome-Based Selection and Characterization of *Fusarium circinatum*-Specific Sequences

**DOI:** 10.1534/g3.115.025817

**Published:** 2016-02-17

**Authors:** Mkhululi N. Maphosa, Emma T. Steenkamp, Brenda D. Wingfield

**Affiliations:** * Department of Genetics, Forestry and Agricultural Biotechnology Institute, University of Pretoria, Faculty of Natural and Agricultural Sciences, 0028 South Africa; † Department of Microbiology and Plant Pathology, Forestry and Agricultural Biotechnology Institute, University of Pretoria, Faculty of Natural and Agricultural Sciences, 0028 South Africa

**Keywords:** pitch canker, diagnostic candidates, unique, genes

## Abstract

*Fusarium circinatum* is an important pathogen of pine trees and its management in the commercial forestry environment relies largely on early detection, particularly in seedling nurseries. The fact that the entire genome of this pathogen is available opens new avenues for the development of diagnostic tools for this fungus. In this study we identified open reading frames (ORFs) unique to *F. circinatum* and determined that they were specific to the pathogen. The ORF identification process involved bioinformatics-based screening of all the putative *F. circinatum* ORFs against public databases. This was followed by functional characterization of ORFs found to be unique to *F. circinatum*. We used PCR- and hybridization-based approaches to confirm the presence of selected unique genes in different strains of *F. circinatum* and their absence from other *Fusarium* species for which genome sequence data are not yet available. These included species that are closely related to *F. circinatum* as well as those that are commonly encountered in the forestry environment. Thirty-six ORFs were identified as potentially unique to *F. circinatum*. Nineteen of these encode proteins with known domains while the other 17 encode proteins of unknown function. The results of our PCR analyses and hybridization assays showed that three of the selected genes were present in all of the strains of *F. circinatum* tested and absent from the other *Fusarium* species screened. These data thus indicate that the selected genes are common and unique to *F. circinatum*. These genes thus could be good candidates for use in rapid, in-the-field diagnostic assays specific to *F. circinatum*. Our study further demonstrates how genome sequence information can be mined for the identification of new diagnostic markers for the detection of plant pathogens.

*Fusarium circinatum* is the causal agent of pitch canker, which is an economically important disease of pines (Wingfield *et al.* 2008). The first known incidence of an epidemic caused by this fungus occurred in the south-eastern United States in 1946 (Gordon *et al.* 2001), but the pathogen has since spread worldwide and is responsible for devastating forestry industry losses (Viljoen *et al.* 1994; Gordon *et al.* 2001; Wingfield *et al.* 2002, 2008; Landeras *et al.* 2005; Carlucci *et al.* 2007; Alonso and Bettucci 2009; Bragança *et al.* 2009; Steenkamp *et al.* 2012; Pfenning *et al.* 2014). In regions where the pathogen occurs, it often also has a complicated life history and distribution that could not have been easily predicted. In South Africa, for example, the pathogen initially appeared to be confined to nurseries where it caused severe root disease on pine seedlings and cuttings (Viljoen *et al.* 1994; Wingfield *et al.* 2008), while it emerged as a plantation pathogen only recently (Coutinho *et al.* 2007; Steenkamp *et al.* 2014) by affecting established or mature pine trees in the Eastern Cape, Western Cape, and KwaZulu Natal Provinces (Britz *et al.* 2005; Coutinho *et al.* 2007; Steenkamp *et al.* 2014; Santana *et al.* 2015).

The global spread of *F. circinatum* could be attributed to trade in seeds while the spread from nurseries to plantations is probably the consequence of practices that involve the planting of contaminated or infected seedlings (Wingfield *et al.* 2008). Therefore, a major challenge facing forestry industries has been the detection of the pathogen in plant growth media and in plant tissues especially during the early stages of infection. However, one of the most significant hurdles in terms of early detection has been the lack of rapid, in-the-field pathogen detection tools. The currently available quantitative real-time PCR methodologies (Schweigkofler *et al.* 2004; Ioos *et al.* 2009; Dreaden *et al.* 2012) all utilize expensive and sophisticated equipment that are not practically and economically feasible for routine use in nurseries and field stations. Alternative tools such as the DNA-based loop-mediated isothermal amplification (LAMP) method (Tomita *et al.* 2008) and antigen-based enzyme-linked immunosorbent assay (ELISA) test kits (Gan *et al.* 1997) would be much more appropriate for in-the-field detection, but have not yet been developed for the pitch canker pathogen.

The development of diagnostic assays based on technologies such as LAMP and ELISA is dependent on the availability of pathogen-specific targets to allow unambiguous identification of *F. circinatum*. In the case of LAMP, the DNA target region should ideally span an area not exceeding 200 bp specific to the genome of *F. circinatum* (Notomi *et al.* 2000; Tomita *et al.* 2008), while the ELISA targets should represent antigenic proteins with epitopes specific to the pathogen (Gan *et al.* 1997). However, the available diagnostic tools for this fungus were mostly developed based on known taxonomic markers and accordingly rely on polymorphisms within highly conserved and/or noncoding DNA regions (Steenkamp *et al.* 1999; Schweigkofler *et al.* 2004; Ioos *et al.* 2009; Dreaden *et al.* 2012), which would not be suitable for LAMP purposes or for developing ELISA tools.

Increased access to whole genome sequence information for fungal pathogens has opened up the possibility of mining these genomes for suitable targets to use in diagnostics. The genome sequences for various *Fusarium* species have been determined previously and are in the public domain; *e.g.*, the *Fusarium* Comparative Sequencing Project (Broad Institute of Harvard and MIT; http://www.broadinstitute.org) and the National Center for Biotechnology Information (NCBI; http://www.ncbi.nlm.nih.gov). This is also true for the pitch canker fungus (Wingfield *et al.* 2012) and its close relatives *F. verticillioides* (*Fusarium* Comparative Sequencing Project) and *F. fujikuroi* (Wiemann *et al.* 2013). Although comparisons among these genomes have revealed high levels of synteny, various chromosomal regions in these fungi have been suggested to be strain- or species-specific (Wiemann *et al.* 2013; De Vos *et al.* 2014). The overall goal of this study was, therefore, to explore the possibility of using genome-based information to identify targets that would be suitable for future development of diagnostic methods based on technologies such as LAMP and ELISA. Our first aim was to analyze the protein-coding component of the *F. circinatum* genome against those of other *Fusarium* species in public databases to identify genes unique to the pitch canker fungus. We then characterized the identified sequences in terms of the proteins they encode, as well as the cellular localization and antigenicity of the inferred proteins. Finally, genes that were apparently specific to *F. circinatum* and that could potentially encode products unique to this fungus were then evaluated for their distribution among isolates of *F. circinatum* and their absence in other species of *Fusarium*, particularly those such as *F. proliferatum* (Stępień *et al.* 2011) and *F. oxysporum* (Fravel *et al.* 2003) which often occur in the same environment as the pitch canker fungus. This study will thus provide the foundation for future development of highly specific diagnostic assays for this important pathogen, both in terms of potential gene targets and the methodologies to identify suitable diagnostic markers.

## Materials and Methods

### Screening of the F. circinatum genome to identify species-specific genes

In this study, the genome sequence information for one strain (FSP34) of *F. circinatum* was used (Wingfield *et al.* 2012). Genome data and predicted protein sequences of *F. oxysporum*, *F. graminearum*, and *F. verticillioides* were obtained from the Broad Institute’s *Fusarium* Comparative Sequencing Project. The genomic data of *F. fujikuroi* that were generated by Wiemann *et al.* (2013) were obtained from the authors. A nucleotide database and a protein database of all these genomes were created on CLC Main Workbench 5.7 (CLC bio A/S). This platform was then used to search for homologs of the *ca*. 15,000 putative genes of *F. circinatum* (Wingfield *et al.* 2012) in the genomes of these other fungi by making use of BLASTn and a word size of 11. In a similar way, the protein sequences encoded by the screened genes were then analyzed on the protein database using BLASTp searches to identify potentially unique proteins in *F. circinatum*. All the identified genes were then screened against the nucleotide and protein sequences databases at the NCBI, using BLASTn and BLASTp searches. For the purposes of this study, unique open reading frames (ORFs) were defined as those showing less than 50% nucleotide sequence identity and encode for proteins returning less than 30% positive amino acid identity from all screened databases.

Putative unique ORFs or ORFs that are potentially specific to *F. circinatum* were subjected to BLASTx and tBLASTn analyses using the search engines and databases of the Broad Institute and NCBI to characterize the potential protein products coded for by these putative genes. All putative genes that potentially coded for protein sequences similar to sequences available in either of these public databases were eliminated from our set of ORFs that are potentially unique to *F. circinatum*.

### In silico characterization of possible F. circinatum-specific genes

To predict functions for the *F. circinatum*-specific candidate genes, their inferred amino acid sequences were analyzed on the following databases: Pfam (Punta *et al.* 2012) to determine which protein family they belong to; conserved domains (CDD) (Marchler-Bauer *et al.* 2011) to deduce any conserved domains they might encode; and Simple Modular Architecture Research Tool (SMART) (Letunic *et al.* 2012) to ascertain the arrangement of different domains (where applicable). To predict the cellular localization of the putative proteins, the following programs were used: SignalP (Dyrløv Bendtsen *et al.* 2004) to predict any signal peptides within the first 70 amino acids of the protein sequence; and WoLF PSORT (Horton *et al.* 2007) to predict subcellular localization. To evaluate if the proteins could be applicable in an immune assay such as ELISA, VaxiJen (Doytchinova and Flower 2007) was used to predict antigenicity. To determine if there could be paralogs within the *F. circinatum* genome we analyzed the ORF sequences against the *F. circinatum* genomic data using the BLASTn function on CLC Bio workbench. We further analyzed the unique candidate sequences against the available *F. circinatum* RNA sequence data (Wingfield *et al.* 2012) to ascertain the evidence of expression.

### Evaluating the specificity of the identified ORFs to F. circinatum

PCR primers were designed as close as possible to the beginning and end of the predicted ORFs by making use of Primer Premier (Abd-Elsalam 2003). These primers (Table 1) were then used to amplify the genes in a set of *F. circinatum* isolates (Table 2). These were specifically chosen to span the known diversity of the fungus, as reported in various studies on its population biology (Viljoen *et al.* 1997; Wikler and Gordon 2000; Steenkamp *et al.* 2014). We also included a set of other *Fusarium* species available in our culture collection in these screenings to evaluate the occurrence of the identified genes in taxa other than the pitch canker pathogen (Table 2). Although this second isolate set included a number of *Fusarium* species, those commonly encountered in pine-based forestry environments were emphasized. Therefore, various isolates were specifically chosen to span a broad diversity in each of *F. oxysporum* and *F. proliferatum*.

**Table 1:** Primers used in this study indicating different annealing temperatures for each primer pair

*F. circinatum* Gene	Name	Sequence 5′-3′	Annealing Temperatures
FCIRG_14470	FCIRG_14470F	CCTCTTCCGCCTCAACTA	55
	FCIRG_14470R	GAGCCGTTTAGCGACCTG	
FCIRG_06550	FCIRG_06550F	CCCTCCCAGCAACCACCG	57
	FCIRG_06550R	CGACCGTTTCCTGGCTGACC	
FCIRG_06217	FCIRG_06217F	AGAGGTCCCAGTAGCAGTAG	54
	FCIRG_06217R	GCACCTTGTCTTCCTCGG	
FCIRG_05181	FCIRG_05181F	CGCAGACGCTGAAGAAAA	57
	FCIRG_05181R	TGGCAGGTTGACAGTGAAAT	
FCIRG_10575	FCIRG_10575F	TCTCGGAATAGGTCTTGTATCAGC	58
	FCIRG_10575R	CCTGGCGAGGCGACATTAGC	

**Table 2:** Fungal isolates and species used in this study as well as their hosts and geographic origins

Isolates*^a^*	Species	Host and Origin*^b^*
CMWF530, CMWF1799, CMWF1800, CMWF1801, CMWF1802, CMWF1803	*F. circinatum*	*Pinus patula*, Mexico, Hildalgo
CMWF550	*F. circinatum*	*Pinus leiophylla*, Mexico, North-central Michoacan
CMWF567	*F. circinatum*	*Pinus douglasiana*, Mexico, Jalisco
CMWF1804	*F. circinatum*	*Pinus greggii*, Mexico, Laguna Atezca
CMWF39, CMWF30, CMWF45	*F. circinatum*	*Pinus patula*, South Africa, Mpumalanga
CMWF56	*F. circinatum*	*Pinus greggii*, South Africa, Mpumalanga
CMWF497	*F. circinatum*	*Pinus patula*, South Africa, Mpumalanga
CMWF538, CMWF513, CMWF659, CMWF674	*F. circinatum*	*Pinus radiata*, South Africa, Western Cape
CMWF350	*F. circinatum*	*Pinus radiata*, USA, California
CMWF968, CMWF1002	*F. oxysporum*	*Syzygium cordatum*, South Africa, Gauteng
CMWF915, CMWF927	*F. oxysporum*	*Syzygium cordatum*, South Africa, KwaZulu Natal
CMWF940	*F. oxysporum*	*Syzygium cordatum*, South Africa, Western Cape
CMWF985	*F. oxysporum*	*Syzygium cordatum*, South Africa, Western Cape
CMWF978	*F. pallidoroseum*	*Syzygium cordatum*, South Africa, KwaZulu Natal
CMWF948, CMWF898	*F. proliferatum*	*Syzygium cordatum*, South Africa, Gauteng
CMWF1155, CMWF1161	*F. proliferatum*	*Syzygium cordatum*, South Africa, KwaZulu Natal
CMWF1182	*F. proliferatum*	*Syzygium cordatum*, South Africa, Western Cape
CMWF1005	*F. solani*	*Syzygium cordatum*, South Africa, Western Cape
CMWF1147	*F. solani*	*Syzygium cordatum*, South Africa, KwaZulu Natal
CMWF1474, CMWF1475	*F. subglutinans*	*Zea mays*, USA, Illinois

a CMWF refers to the *Fusarium* culture collection of the Forestry and Agriculture Biotechnology Institute, FABI, University of Pretoria, Pretoria, South Africa.

b The isolates of *F. circinatum* were all reported from previous studies where those from Mexico and California were used by Wikler and Gordon (2000), while those from the Western Cape and Mpumalanga provinces of South Africa were respectively reported by Steenkamp *et al.* (2014) and Viljoen *et al.* (1997). The representatives of *F. subglutinans* came from the study of Steenkamp *et al.* (2001). All of the isolates from *Syzigium cordatum* originated from a previous survey of the diversity of *Fusarium* species associated with this host in South Africa (Kvas *et al.* 2008; E. Steenkamp, unpublished data).

For these PCR-based analyses, we used 25-μl reaction mixtures consisting of 2.5 mM of each dNTP, 2.5 mM MgCl_2_, 10 μM of each primer, 100 ng template DNA, 0.03U *Taq* DNA polymerase, and reaction buffer (Roche). The PCR cycling conditions were as follows: initial denaturation hold at 94° for 5 min, 30 cycles of denaturation at 94° for 30 sec, annealing for 30 sec (see Table 1 for specific temperatures), and elongation at 72° for 30 sec, one hold for elongation at 72° for 7 min, followed by a final hold at 4°. The samples were analyzed using 2% agarose gel electrophoresis (Sambrook *et al.* 1989) using gel red as a DNA indicator and a 100 bp ladder (Promega) as a size marker.

All amplicons were purified using the Invitek PCR clean up kit and then sequenced in both directions using the original PCR primers. For this purpose the Big Dye kit (Applied Biosystems, Foster City, CA) and an ABI PRISM 3100 Autosequencer (Applied Biosystems) at the University of Pretoria’s sequencing facility were used. All sequence traces were analyzed and assembled into contigs using CLC Bio workbench, after which sequence alignments were conducted using ClustalW in Mega version 5 (Tamura *et al.* 2011). Sequences derived from *F. circinatum* isolates were analyzed against each other to check for variations and sequences from other *Fusarium* species were compared to the *F. circinatum* sequences to check for similarities.

We used dot blot hybridization assays to screen for the presence of the identified candidate genes in each of the isolates included in the study. These assays were also used to resolve instances where PCR resulted in no amplification and/or multiple amplicons that could not be sequenced. For these assays, we utilized Roche’s DIG (digoxigenin) High Prime DNA Labeling and Detection Kit (Roche, Manheim, Germany). Genomic DNA of the fungal isolates (Table 2) was blotted onto positively charged nylon membranes and hybridized at 42° with the respective random primed DIG-labeled amplicons of *F. circinatum* isolate FSP34 (*i.e.*, the labeled amplicon for each of the candidate genes was hybridized to the genomic DNA of each of the respective isolates). All hybridizations and detections were conducted according to the manufacturer’s instructions.

### Data availability

All the genome sequences used in this study are available without restriction.

## Results

### Screening of the F. circinatum genome to identify species-specific genes

BLASTn analyses against the genomic database of *F. oxysporum*, *F. graminearum*, *F. verticillioides*, and *F. fujikuroi* returned 411 *F. circinatum* ORFs that were <50% similar to those of the other fungi. This set of ORFs also did not include smaller genes (<450 bp) that would encode proteins less than 140 amino acids long as their limited size might complicate detection assays based on ELISA technologies. BLASTp analyses using the 411 ORF sequences resulted in the identification of 214 predicted *F. circinatum* proteins that showed <30% amino acid sequence similarity to those in the other *Fusarium* genomes. Screening of these 214 ORFs against NCBI’s database identified three ORFs that were more than 50% similar at the nucleotide level to other genes in the database. After excluding these ORFs, screening of the predicted amino acid sequences for the remaining 211 ORFs against NCBI’s protein database returned 36 putative proteins that shared <30% amino acid similarity to other proteins in the database (Table 3). A final screening of these 36 ORFs against the NCBI and Broad Institute databases using BLASTx and a tBLASTn confirmed that they all represented potentially unique sequences in the pitch canker fungus.

**Table 3:** Genes that are potentially unique to *F. circinatum* indicating gene sizes, protein sizes, and number of introns as per information derived from the *F. circinatum* genome annotation

Name of Gene in FSP34	Gene Size	Predicted Protein Size	Expression Values*^a^*	Number of Introns
FCIRG_01122	819	166	—	1
FCIRG_12049	2631	479	9.33	6
FCIRG_07223	3057	944	4.42	3
FCIRG_06393	712	206	17.23	2
FCIRG_00789	2520	375	7.47	4
FCIRG_14829	1022	290	7.04	4
FCIRG_05207	530	169	2.68	1
FCIRG_05759	1863	412	46.49	2
FCIRG_03368	708	219	5.86	2
FCIRG_08620	945	249	3.64	3
FCIRG_03489	3179	1038	—	1
FCIRG_14907	537	136	—	3
FCIRG_14908	647	144	26.17	3
FCIRG_12843	1632	544	4.46	—
FCIRG_15130	1139	349	—	2
FCIRG_12122	2611	733	8.04	4
FCIRG_10746	1334	189	16.78	1
FCIRG_14470	1227	409	1.11	—
FCIRG_13499	630	174	3.90	2
FCIRG_13677	1011	337	—	—
FCIRG_06550	1284	428	0.53	—
FCIRG_02584	3390	795	2.10	2
FCIRG_06217	820	263	44.84	1
FCIRG_10116	4186	1045	11.75	5
FCIRG_06189	2734	849	6.34	3
FCIRG_05800	1918	551	1.10	4
FCIRG_03074	2311	579	0.39	9
FCIRG_09402	508	155	—	1
FCIRG_05181	589	190	0.79	1
FCIRG_03107	724	228	3.65	2
FCIRG_10765	1982	375	—	6
FCIRG_04655	1706	259	2.34	2
FCIRG_10144	1484	439	11.21	3
FCIRG_02555	2585	540	—	8
FCIRG_09038	949	173	1.74	3
FCIRG_10575	486	159	0.47	1

The expression values were extracted from the available RNA sequence data.

a Expression values derived from RNA sequence data in reads per kilobase per million (RPKM).

### In silico characterization of possible F. circinatum-specific genes

Of the 36 putative genes potentially unique to *F. circinatum*, 19 encode proteins with known domains (Table 4) and 17 encode proteins of unknown function (Table 5). SignalP predicted that three of the putative proteins had signal peptides and were also predicted to be extracellular proteins by WoLF PSORT. Some putative proteins were predicted to represent mitochondrial proteins, but these were likely exported to this organelle as no significant hits were obtained when comparing the ORFs against the *F. circinatum* mitochondrial genome data (Fourie *et al.* 2013), thus confirming that all of the 36 ORFs are encoded on the nuclear genome. Twenty-four putative proteins were predicted to be potentially antigenic, suggesting that they are good candidates for an immune-based diagnostic assay. No paralogs of any of these ORFs were identified in the *F. circinatum* genomic data and we found evidence of expression in *F. circinatum* for 28 of the ORFs (Table 3).

**Table 4:** *F. circinatum* potentially unique candidate genes with known putative domains, indicating putative protein families and domains, the top predicted subcellular localization, and whether proteins are antigens or nonantigens

Name of Gene in FSP34	Pfam*^a^*	CDD*^b^*	SignalP*^c^*	WoLF PSORT*^d^*	Vaxijen*^e^*
FCIRG_07223	Oxidored_FMN	OYE_like_FMN	N	cyto	Nonantigen
		TIM_phosphate_binding superfamily			
		NAD_binding_8 superfamily			
		NemA			
FCIRG_00789	Fungal_trans_2	Fungal_trans_2 superfamily	N	plas	Antigen
	RTA1	RTA1 superfamily			
FCIRG_05207	RR_TM4-6		N	cyto_nucl	Nonantigen
	DUF4337				
	IFP_35_N				
FCIRG_05759	DUF2935		N	cyto_nucl	Antigen
FCIRG_03368	DPBB_1	PAT1	Y	extr	Antigen
FCIRG_03489	TcdA_TcdB_pore	TcdA_TcdB_pore superfamily	N	mito	Antigen
	Pfam-B_4370				
	Pfam-B_8938				
FCIRG_14908	HET	HET superfamily	N	mito	Antigen
FCIRG_12843	Lysine_decarbox	Lysine_decarbox superfamily	N	cyto	Antigen
FCIRG_15130	Pfam-B_12758		N	nucl	Antigen
FCIRG_12122	MMR_HSR1	Ras_like_GTPase superfamily	N	nucl	Nonantigen
FCIRG_13499	Elong_Iki1		Y	extr	Nonantigen
FCIRG_10116	Peptidase_S8	Peptidases_S8_S53	N	nucl	Antigen
FCIRG_06189	Pfam-B_19120	ZnF_C2HC	N	nucl	Antigen
FCIRG_05800	Pfam-B_360	Abhydrolase_6	N	nucl	Antigen
FCIRG_03074	DDR	NBD_sugar-kinase_HSP70_actin superfamily	N	cysk	Antigen
FCIRG_10765	MFS_1	HpaX	N	plas	Nonantigen
FCIRG_04655		tail_TIGR02242 superfamily	N	cyto_nucl	Antigen
FCIRG_02555	Aldedh	NBD_sugar-kinase_HSP70_actin superfamily	N	cyto	Antigen
FCIRG_09038	ADIP		N	nucl	Antigen

a Protein family as predicted by the program Pfam.

b Conserved domains as predicted from the conserved domain database.

c Presence (Y) or absence (N) of signal peptides as predicted by the program SignalP.

d Top predicted subcellular localization of the putative proteins as predicted by the program WoLF PSORT.

e Predicted antigenicity or nonantigenicity of the putative proteins as predicted by the program Vaxijen.

**Table 5:** *F. circinatum* potentially unique candidate genes with no currently known protein motifs indicating top hits on subcellular localization, signal peptides (N, not present and Y, present) and whether proteins are antigens or nonantigens

Name of Gene in FSP34	SignalP	WoLF PSORT	VaxiJen
FCIRG_01122	N	cyto_nucl	Nonantigen
FCIRG_02584	N	cyto	Antigen
FCIRG_03107	Y	extr	Nonantigen
FCIRG_05181	N	cyto	Antigen
FCIRG_06217	N	mito	Antigen
FCIRG_06393	N	nucl	Antigen
FCIRG_06550	N	extr	Antigen
FCIRG_08620	N	nucl	Antigen
FCIRG_09402	N	cyto_nucl	Nonantigen
FCIRG_10144	N	mito	Nonantigen
FCIRG_10575	N	mito	Antigen
FCIRG_10746	N	nucl	Nonantigen
FCIRG_12049	N	nucl	Antigen
FCIRG_13677	N	nucl	Nonantigen
FCIRG_14829	N	nucl	Antigen
FCIRG_14907	N	nucl	Nonantigen
FCIRG_14470	N	extr	Antigen

See Table 4 for description of the various entries.

### Evaluating the specificity of the identified ORFs to F. circinatum

The 17 genes that encode putative proteins without any known domains were regarded as good candidates for diagnostics. This is because their use might eliminate cross-reactivity associated with the use of proteins with conserved domains that can present the same epitopes. Among the 17 ORFs encoding proteins with no known domains, we selected five for which we found evidence for expression and that potentially encode antigenic proteins. Therefore, primers were designed to amplify the five *F. circinatum* genes FCIRG_14470, FCIRG_06550, FCIRG_06217, FCIRG_05181, and FCIRG_10575. Three primer sets designed for the genes FCIRG_14470, FCIRG_05181, and FCIRG_10575 resulted in amplicons of the expected size in all tested isolates of *F. circinatum*. Sequence analyses of the FCIRG_05181 amplicons revealed single nucleotide polymorphisms among different isolates of *F. circinatum*, while no differences were observed in FCIRG_10575 and FCIRG_14470. The primer set designed for FCIRG_06217 amplified different sized amplicons in the various *F. circinatum* strains. Sequence analyses of these amplicons revealed that the observed polymorphism is due to various indels (20−115 bp) in different *F. circinatum* isolates. The PCRs with the primers designed for FCIRG_06550 failed to generate amplicons in some *F. circinatum* isolates (Table 6). These findings were confirmed by the results of the dot blot hybridization assays, where positive hybridization was observed for all of the reactions with the probes for FCIRG_14470, FCIRG_06217, FCIRG_05181, and FCIRG_10575. Reactions with the probe for FCIRG_06550 only showed positive hybridization for those isolates from which the corresponding amplicon could be generated.

**Table 6:** Summary of PCR amplification of the five selected genes in different strains of *F. circinatum*

Isolates	FICIRG_06217	FCIRG_06550	FCIRG_10575	FCIRG_05181	FCIRG_14470
CMWF30	+	+	+	+	+
CMWF39	+	+	+	+	+
CMWF45	+	+	+	+	+
CMWF56	+	—	+	+	+
CMWF350	+	+	+	+	+
CMWF497	+	+	+	+	+
CMWF538	+	+	+	+	+
CMWF513	+	—	+	+	+
CMWF659	+	+	+	+	+
CMWF674	+	+	+	+	+
CMWF530	+	—	+	—	—
CMWF550	+	+	+	+	+
CMWF560	+	—	+	+	+
CMWF567	+	+	+	+	+
CMWF1221	+	+	+	+	+
CMWF1799	+	+	+	+	+
CMWF1800	+	+	+	+	+
CMWF1801	+	+	+	+	+
CMWF1802	+	+	+	+	+
CMWF1803	+	+	+	+	+
CMWF1804	+	+	+	+	+

Summary of PCR results indicating successful amplification (+) and no amplicon obtained (—). Mexican isolate CMWF530 gave inconsistent results.

**Figure 1:**
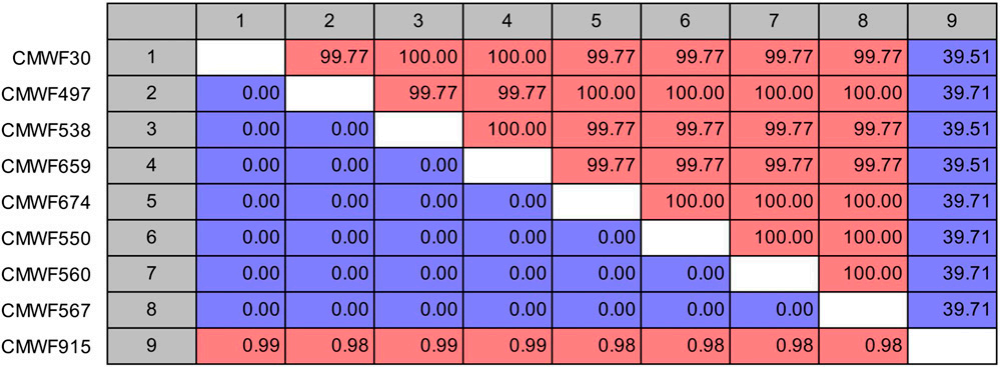
Pairwise comparison of FCIRG_05181 amplicon sequences from different strains of *F. circinatum* (CMWF30, CMWF497, CMWF538, CMWF659, CMWF674, CMWF550, CMWF560, and CMWF567) and *F. oxysporum* isolate (CMWF915). Percentage similarity is shown above the diagonal and Jukes–Cantor corrected distances are shown below the diagonal.

No corresponding amplicons of the expected size were amplified using any of the five primers pairs in the other *Fusarium* species tested. Although not within the expected size range, amplicons were obtained in some *Fusarium* species. Primers for FCIRG_10575 resulted in multiple-sized amplicons with most of the *Fusarium* species tested, and no sequence analysis was done on its amplicons. Sequencing of the amplicons obtained with the primers for FCIRG_06550, FCIRG_05151, FCIRG_14470, and FCIRG_06217 from the non-*F. circinatum* isolates showed that they were all different from those of *F. circinatum*. Sequence comparison of the FCIRG_05181 amplicon obtained from *F. oxysporum* with *F. circinatum* also resulted in <50% identity (Figure 1). Based on our parameters for defining unique ORFs, none of the sequences from the other species (including *F. oxysporum*) was therefore regarded as similar or homologous to those of *F. circinatum*. These findings further corresponded with the results of the dot blot hybridization assays, which suggested that FCIRG_05151, FCIRG_14470, FCIRG_06217, and FCIRG_06550 were absent from all of the non-*F. circinatum* isolates tested. The only exception was FCIRG_10575, which appeared to be present in both of the tested *F. subglutinans* isolates.

## Discussion

In this study, we utilized a genome-based *in silico* approach to identify and characterize a set of genes that are potentially unique to *F. circinatum*. Although it is possible that we might have excluded suitable gene targets during the initial identification phase of the process, our use of >50% and >30% sequence similarity cut-off values, at the respective DNA and protein levels, ensured that the genes or ORFs identified in this fungus encode products that are quite distinct from other proteins. In other words, strongly conserved genes with homologous sequences in related fungi were excluded to limit the possibility of unwanted cross-reactivity in diagnostic assays. For example, a LAMP assay utilizes six primers targeting eight regions within a DNA fragment of between 130 bp and 200 bp; and for it to be unambiguous, all the primers have to be specific to the target organism (Notomi *et al.* 2000). Such cross-reactivity can also occur in an immune-based assay such as ELISA which utilizes the interactions between an antibody and epitopes on an antigen; and homologous proteins that potentially have similar folding patterns could present similar epitopes that would allow cross-reaction with antibodies. Our relatively conservative approach for identifying genes or ORFs unique to *F. circinatum* thus facilitated compilation of a list of putative gene targets that are sufficiently variable to ultimately allow for their potential use in the diagnostics of this pathogen.

Among the set of 36 ORFs potentially unique to the pitch canker fungus, 17 encode proteins with obscure features (POFs) (Armisén *et al.* 2008) that lack known and defined motifs or domains. Arguably, these ORFs would represent good candidates for diagnostics because of their apparent uniqueness and lack of domains common to other organisms. Although all 17 of these ORFs appear to be transcribed and 10 are predicted to be antigenic, more work is, however, needed to fully understand their expression and the types of proteins they encode, before utilizing them for immune-based procedures. The ideal candidates for an immune-based assay would be genes that are constitutively expressed in all the life stages of the pathogen, while their protein products are stable and easily accessible or extractable (Gan *et al.* 1997).

The other 19 ORFs that are potentially unique to *F. circinatum* encode proteins involved in a range of different processes. These include cellular division (FCIRG_03368) (Wang *et al.* 1996), growth (FCIRG_12122) (Callebaut *et al.* 2001), and maintenance (FCIRG_10765) (Pao *et al.* 1998), as well as host colonization (FCIRG_10116, FCIRG_05800, and FCIRG_00789) (Soustre *et al.* 1996; Suárez *et al.* 2007; Carr and Ollis 2009). Some of these ORFs also encode substrate-transforming proteins (FCIRG_03079, FCIRG_02555, FCIRG_12843, and FCIRG_07223) (Williams and Bruce 2002), while others encode products potentially involved in transcription (FCIRG_00789) (Shelest 2008) and nonself recognition (FCIRG_14908) (Espagne *et al.* 2002). One of the identified ORFs encoded the TcdA/TcdB pore motif (FCIRG_03489) of the *Clostridium difficile* toxin A and toxin B pore-forming region (Qa’Dan *et al.* 2000). Clostridial toxins A and B are a class of virulence factors that cause serious diseases in mammals (Qa’Dan *et al.* 2000) and their occurrence in fungi and effects on plants has not been reported.

All 36 ORFs were compared against the *F. circinatum* mitochondrial genome assembly data (Fourie *et al.* 2013) to check if any of them could represent mitochondrial genes. No significant hits were obtained indicating that these were all nuclear genes. Roughly 1% of mitochondrial proteins are typically encoded by the mitochondrial genome while the rest are encoded on the nuclear genome (Pfanner and Geissler 2001; Schmidt *et al.* 2010). As a result, the large majority of mitochondrial proteins are synthesized as precursor proteins in the cytoplasm and imported into the organelle (Schmidt *et al.* 2010). Our results thus suggest that at least four of the ORFs (FCIRG_03489, FCIRG_14908, FCIRG_06217, and FCIRG_10144) apparently unique to *F. circinatum* encode for proteins that are transported in a similar way into the mitochondrion. It would be interesting to understand exactly how they function in this cellular compartment and whether or not they potentially convey unique mitochondrial traits to the pathogen.

The available *F. circinatum* genome harbored no detectable paralogs of the 36 unique ORFs and all of them, therefore, appeared to represent single copy nuclear genes. Although multi-copy genes are usually regarded as good candidates for DNA-based diagnostics because of enhanced sensitivity compared to single copy genes (Ioos *et al.* 2009), there are limitations associated with their use in this context. Some of the notable limitations include intragenomic heterogeneity (Morandi *et al.* 2005) that could lead to misidentification of species (Graf 1999). Single copy genes, however, can often be quite useful as diagnostic markers (Álvarez *et al.* 2008) because they are less likely to be subject to complexities related to intragenomic polymorphisms (*i.e.*, differences among the paralogs of a gene) (Simon and Weiß 2008).

By making use of a PCR-based approach and dot blot hybridization assays, we evaluated the ubiquitous presence of a subset of five unique ORFs in a diverse collection of *F. circinatum* isolates. These assays indicated that homologs for four of the five genes tested (*i.e.*, FCIRG_14470, FCIRG_06217, FCIRG_05181, and FCIRG_10575) were present in all of the genetically and geographically diverse *F. circinatum* isolates evaluated, while only some isolates of this fungus appear to harbor a homolog of FCIRG_06550. Through sequence analysis, we also showed that the amplified products corresponded to the original FSP34 sequences, although we did observe various single nucleotide polymorphisms (FCIRG_05181) and indels (FCIRG_06217) among the isolates. Therefore, based on their ubiquitous presence in *F. circinatum*, at least four of the tested genes represent potential candidates for the development of rapid in-the-field diagnostic assays for this pathogen.

For diagnostic assays to be reliable, they should ideally produce unambiguous and conclusive diagnoses. In other words, if a specific marker region is used, it should be present in all individuals of the focal species to avoid recording false negatives; the results of our screenings with the diverse set of *F. circinatum* isolates allowed evaluation of this issue. However, the ideal diagnostic marker should also be absent from all nonfocal species to avoid recording false positives. This aspect was evaluated by screening a set of non-*F. circinatum* isolates for the presence/absence of the target genes. The PCR and dot blot hybridization assays showed that none of the evaluated isolates encodes a homolog of any of the five genes tested. The only exception was for FCIRG_10575, which appeared to be also present in *F. subglutinans*, which is closely related to *F. circinatum* (Kvas *et al.* 2009). Although *F. subglutinans* is unlikely to be encountered in the commercial forestry environment (Kvas *et al.* 2009; Leslie and Summerell 2006), the fact that it apparently harbors a homolog of FCIRG_10575 points toward the potential presence of the gene in other species of the so-called “American Clade” of the *Gibberella fujikuroi* complex of which *F. circinatum* is also a member (Kvas *et al.* 2009). This considerably detracts from the potential value of gene FCIRG_10575 as a diagnostic marker because its use might lead to recording of false positives when non-*F. circinatum* members of the “American Clade” of the complex are encountered.

Taken together, these findings suggest that the four ORFs found in all of the *F. circinatum* isolates examined represent members of the so-called core genome of the fungus (Hsiang and Baillie 2005). However, our findings also indicated that only those core genome components not shared with those of other species would be useful for the development of robust diagnostic assays (*i.e.*, the use of core genome regions that overlap with those of other species would lead to false negatives). The ORF that was absent from some *F. circinatum* isolates is potentially lineage-specific, forming part of its so-called accessory genome (Croll and McDonald 2012). Although the genes encoded on this component of the fungal genome is often associated with adaptive properties such as virulence and/or pathogenicity (Croll and McDonald 2012), their use in diagnostics is limited due to the high likelihood of recording false negatives.

Here we showed that comparative genomic studies allow for the identification of species-specific traits that can be used to identify a taxon. Species-specific traits might be genomic regions that are unique and fixed to a particular species or strongly modified compared to homologous loci in close relatives. In this study, genomic regions that are unique to *F. circinatum* and are fixed in different strains of the pitch canker fungus were identified. Although care should be taken to avoid regions characterized by high levels of intraspecific polymorphism, these genomic regions appear to be good candidates for use as targets in a *F. circinatum* species-specific diagnostic assay. However, lack of functional annotation of these genes makes it very difficult to infer or speculate on their significance within the *F. circinatum* genome. Tracing the origins of these genes will also go a long way in validating any diagnostic assay that may be developed based on them. Nevertheless, the findings of this study thus represent a fundamental resource for the development of diagnostic tool(s) for the pitch canker pathogen as at least three of the gene targets identified could be used to develop rapid methods for in-the-field diagnosis of the pathogen. Our novel approach and the workflow employed can also easily be adapted for identifying species-specific diagnostic markers for other important taxa.
